# Boosting HSA Vaccination with Jujube Powder Modulating Gut Microbiota Favorable for Arginine Metabolism

**DOI:** 10.3390/nu15081955

**Published:** 2023-04-19

**Authors:** Huiren Zhuang, Zhenghuan Yang, Tianhao Chen, Nan Jing, Yalin Zhou, Guoqiang Jiang, Yi Wang, Zhao Wang, Zheng Liu

**Affiliations:** 1 Key Lab of Industrial Biocatalysis, Ministry of Education, Department of Chemical Engineering, Tsinghua University, Beijing 100084, China; zhuanghr22@mails.tsinghua.edu.cn (H.Z.);; 2 Food Science and Technology, National University of Singapore, Singapore 117542, Singapore; 3 MOE Key Laboratory of Protein Science, School of Pharmaceutical Sciences, Tsinghua University, Beijing 100084, China

**Keywords:** vaccination response, ABX, FMT, jujube, gut microbiota

## Abstract

Whereas vaccination is established as one of the most effective and available methods against seasonal flu and holds high potential for many infectious diseases, immune response may differ among individuals and regions. In this study we examined the effects of gut microbiota on vaccination with human serum albumin (HSA) as the model vaccine in C57BL/6J mice. We observed that a two-week antibiotic cocktail (ABX) treatment hampered HSA-specific IgG1 in serum, whereas fecal microbiota transplantation (FMT) restored the gut microbiota impaired by the ABX treatment and consequently increased the proportions of macrophages in the mesenteric lymph nodes (MLNs), plasma cells in the peripheral blood, and HSA-specific immunoglobulin G1 (IgG1) in the serum. A week of daily application of jujube powder (800 mg/kg) to ABX-treated mice achieved a significantly higher HSA-specific IgG1 concentration in the serum compared with the ABX treatment group. Of particular note was that the administration of the jujube powder did not increase the myeloid cells, indicating a different mechanism of vaccination compared with FMT. More interestingly, daily pre-administration of jujube powder (800 mg/kg) to healthy mice one week ahead of vaccination boosted their immune response, as evidenced by the proportion of macrophages in the MLNs, B cells in the spleen, plasma cells and memory B cells in the peripheral blood, and HSA-specific IgG1 concentration in the serum. The 16S rRNA sequencing of gut microbiota revealed that the administration of jujube powder increased the abundance of *Coriobacteriaceae* associated with the metabolism of amino acids. The Kyoto encyclopedia of genes and genomes (KEGG) analysis suggested the altered microbiota is more favorable for arginine and proline metabolism, which may promote macrophages in the MLNs. These results indicate a high potential for boosting vaccination by manipulating gut microbiota with natural products.

## 1. Introduction

Vaccination has been extensively applied against seasonal flu worldwide and holds high potential for infectious diseases. However, and unfortunately, growing evidence has been reported on the variation in vaccination response between individuals and regions. Zimmermann. et al. [1] found, among 212 individuals, that the IgG concentrations induced by seasonal influenza vaccine could vary more than 100-fold. PrabhuDas et al. [2] and Ciabattini et al. [3] observed that the quality and durability of vaccines were poorer in infants and the elderly in comparison with adults. Clark et al. [4] demonstrated that oral rotavirus vaccine (ORV) efficacy was 94% in high-income countries (HICs) after 12 months, whereas for low-income and middle-income countries, the efficacy was 44%. Given the painful experience of COVID-19, it is imperative to develop workable ways to improve vaccination efficiency worldwide.

Gut microbiota may play a vital role in modulating the vaccine immune response. Hagan et al. [5] found that gut microbiota impairment by antibiotics treatment hampered immunoglobulin G1 (IgG1) and IgA responses to the trivalent influenza vaccine (TIV) in individuals with low pre-existing immunity and induced inflammatory signatures through AP-1/NR4A. Using mice models, Lynn et al. [6] demonstrated that antibiotic exposure in early life severely hampered antibody responses to five vaccines, which could be restored by fecal microbiota transplantation (FMT) from healthy mice of the same age. Jason et al. [7] found that flagellin produced by gut microbiota stimulated toll-like receptor 5 (TLR-5) and enhanced plasma-cell differentiation and TIV-specific IgG concentration by provoking macrophages in lymph nodes. Kim et al. [8] discovered that sensing of the gut microbiota is critical for cholera toxin (CT) by means of the nucleotide-binding oligomerization domain containing 2 (Nod2). Kim et al. [9] demonstrated that short-chain fatty acids (SCFAs), fermented from dietary fiber in the gut microbiota, boosted IgA and IgG response by stimulating B-cell metabolism and plasma-cell differentiation. Tang et al. [10] found that the concentration of SCFAs in the feces and serum is positively correlated with vaccination using the BBIBP-CorV vaccine. More recently, Jia et al. [11] demonstrated that pre-existing antibodies in the gut microbiota cross-reacted with SARS-CoV-2, increasing the receptor-binding domain (RBD) binding antibody titers of 28 healthy individuals after vaccination with the BBIBP-CorV vaccine. All these results indicate the possibility of boosting vaccination by modulating gut microbiota.

FMT [6], prebiotics [9,12], probiotics [13], and symbiotics [14] have been tested to regulate gut microbiota for boosting vaccination. Lynn et al. [6] restored gut microbiota to recover the impaired five live and adjuvanted vaccine immune responses using FMT. Cait et al. [12] applied dietary fiber to improve the TIV-specific IgG level in mice but did not find significant enhancement of the humoral response to the seasonal influenza vaccine. Xu et al. [13] found that administration of *Lactobacillus plantarum* to mice boosted the COVID-19 antibody concentration twofold and prolonged neutralization over 6 months. Di Luccia et al. [14] demonstrated that five bacterial strains combined with nutraceuticals enhanced cholera-toxin (CT)-specific IgA in the feces of hyporesponsive mice.

Ziziphus jujube is rich in flavonoids [15], phenolic acids [16], amino acids [17], and polysaccharides [18], and has been widely applied in Chinese traditional medicine. Our previous work demonstrated that the administration of jujube powder increased the abundance of *Bifidobacteria* and enhanced the efficiency of cyclophosphamide on colon cancer by enriching CD8^+^ T cells in the murine tumor environment [19]. Ultrafine jujube powder improved the response of anti-PD-L1 treatment to murine colon cancer in mice by increasing the abundance of *Clostridia*, which are associated with producing SCFAs [20]. All these indicate a high potential of jujube powder in boosting immune response by modulating gut microbiota. In this study, we examined the effects of jujube powder on vaccination using human serum albumin (HSA) as a model vaccine. We determined HSA-specific IgG1 in serum and immune cells (B cells, plasma cells, macrophages, memory B cells, and myeloid cells) in the mesenteric lymph nodes (MLNs). We determined the gut microbiota diversity, composition, and metabolites using 16S rRNA gene sequencing and GC-MS untargeted metabolomics. We found that jujube powder increased the abundance of *Gordonibacter,* which could induce antigen-presenting cell differentiation [21]. The abundance of *Coriobacteriaceae*, which is related to the metabolism of amino acids [22], was enriched by administration of jujube. Kyoto encyclopedia of genes and genomes (KEGG) pathway analysis indicated that jujube powder administration enhanced arginine and proline metabolism, revealing the possible mechanism of promoting macrophages in MLNs [23], consequently stimulating plasma-cell differentiation and IgG production. This study demonstrates that jujube as a prebiotic provides a high potential method to enhance HSA vaccine immune response and suggests a possible mechanism.

## 2. Materials and Methods

### 2.1. Materials

The jujube was purchased from Ruoqiang County, Xinjiang Province; the jujube powder was milled to 1–10 μm with an ultrafine pulverizer (Pls, Jinan, China) at −20 °C for 15 min. Human serum albumin (HSA) was purchased from Sigma-Aldrich (Merck, Darmstadt, Germany). The vaccine adjuvant used was Class B CpG oligonucleotide Murine TLR9 agonist, oligodeoxynucleotide 1826 (ODN1826) (Thermo Fisher, Waltham, MA, USA).

### 2.2. Mice and Treatment

C57BL/6J male mice (6–7 weeks) were purchased from the Vital River Laboratory Animal Technology Co., Ltd. (Beijing, China). The mice were acclimatized in the animal care facilities of Tsinghua University for a week before the animal experimental operation under specific pathogen-free (SPF) conditions. In the ABX-treated mice model, firstly, mice were fed water containing an antibiotic cocktail (ABX) (1 g/L ampicillin, 1 g/L neomycin, 1 g/L metronidazole, and 0.5 g/L vancomycin) for 2 weeks. Then, mice in the ABX+J+HSA group were given a daily oral administration of jujube powder (800 mg/kg) dissolved in sterile water for a week. Mice in the ABX+FMT+HSA group were given daily fecal microbiota transplantation (FMT) from the feces of mice of the same strain and age for 3 days. After that, the mice were intramuscularly (i.m.) injected with HSA (90 μg) and ODN1826 (5 μg) dissolved in 100 μL saline solution. The mice in the control (CTR) group were i.m. injected with an equal volume of saline solution. Each of the CTR, HSA, ABX+HSA, ABX+J+HSA, and ABX+FMT+HSA groups had eight mice.

In the healthy mice model, mice in the J group were given a daily pre-administration of jujube powder (800 mg/kg) dissolved in sterile water for a week, and then they were i.m. injected with HSA (90 μg) and ODN1826 [24] (5 μg) dissolved in 100 μL saline solution. Each of the CTR, J, HSA, and HSA+J groups had 10 mice. Peripheral blood was collected from the mice (30 μL) with 1.5% isoflurane every week through retro-orbital sinus puncture into capillary tubes. The total number of mice used in the experiment was 80. All animal experimental methods were conducted to conform to the guidelines of the Animal Care and Use Committee of Tsinghua University (No. 20-LZ1#).

### 2.3. FMT

Feces were collected from mice of the same age and strain and were stored at −80 °C. The frozen stool samples were defrosted in a 37.5 °C thermostatic water bath for 10 min. The fecal samples were re-suspended in sterile phosphate buffered saline (PBS) 5% (*w*/*v*) and then diluted (200 mg fecal sample into 2 mL volume) and stirred until there were no significant large particles. A 200-mesh sterile mesh sieve was used to remove large particles from the feces, and then the filtrate was passed through 400-mesh and 800-mesh sterile mesh sieves to remove undigested food and smaller particulate matter. The filtrate obtained through the 800-mesh screen was collected in a sterile centrifuge tube. The resuspension was shaked with vortex for 5 min. The suspension was centrifuged at 600× *g* for 5 min to remove the insoluble matter. Each mouse was daily garaged with a 200 μL suspension for 5 days.

### 2.4. DNA Extraction and Bacterial Identification in Stool Samples

Mice were fed with jujube daily for a week. The feces were collected and processed for DNA extraction using the QIAamp DNA Stool Mini Kit (Qiagen, Hilden, Germany) according to standard protocols in www.qiagen.com/HB-1764. They were purified with Gel Extraction Kit of AxyPrep DNA (Axygen, Corning, NY, USA). The PCR products were quantitatively analyzed with the QuantiFluor-ST (Promega, Beijing, China). After that, the fecal DNA was sequenced with the Illumina MiSeq (Majorbio, Shanghai, China) using the standard methods required. The low-quality sequences were removed with the FASTP tool, then the high-quality sequences were clustered to OTUs at 97% identity with the UPARSE tool. The raw sequences of 16S rRNA are acquirable after 18 June 2025 (https://www.ncbi.nlm.nih.gov/) through project ID PRJNA942135.

### 2.5. Bioinformatics Analysis

The gut microbiota alpha diversity was computed and drawn with the Vegan and ggplot2 packages, respectively, in R (version R 3.6.1). The beta diversity and the principal component analysis (PCA) of the different groups were calculated with a mixOmics package. The top represented 5% OTUs of the first component were figured out of the partial least squares discrimination analysis (PLS-DA) result and drawn with R package ggplot2. KEGG enrichment was used to evaluate biological function caused by changes in the gut microbiota metabolites.

### 2.6. Flow Cytometry of Immune Cells in Mesenteric Lymph Nodes (MLNs), Blood, and Spleen

MLNs and spleens were milled with Hank’s buffered solutions to single-cell suspensions with a 70-µm nylon cell strainer. The cells were pelleted by centrifugation (350× *g*) for 5 min and the supernatant aspirated. The whole spleen cells were lysed with 5 mL mixing red-blood lysis buffer (BioLegend, San Diego, CA, USA) for 5 min on ice. The reaction was stopped by adding 20 mL PBS. Then the cells were spun (350× *g*) for 5 min and the supernatant was discarded. Heparinized tubes were used to collect 200 μL of peripheral blood, which was lysed with 2 mL red blood cell lysis buffer for 5 min. on ice; the reaction was stopped by adding 8 mL PBS. Then the cells were spun (350× *g*) for 5 min and the supernatant discarded.

First, the cells were bound with anti-CD16/32 antibody. After that, the cells were stained with mixed antibodies (CD45, CD19, CD11b, CD138, CD27, F4/80, and IgG1) in Hank’s buffered saline solution for 15 min in a dark environment. The proportion of mixed antibodies was the same (5 μL/100 μL Hank’s buffer). The stained cells were washed with 1.5 mL Hank’s buffered saline solution. Then the cells were spun (350× *g*) for 5 min and the supernatant discarded. Then the precipitate was suspended with PBS and analyzed with LSRII cell analyzers (BD Biosciences, Union City, CA, USA). The data was calculated with FlowJo 10.6.2. Cell markers and fluorophore conjugates are shown in Table 1 and Table 2.

### 2.7. HSA-Specific IgG1 in Serum by ELISA

The mice were bled weekly post-HSA vaccination through retro-orbital sinus puncture into tubes. The peripheral blood was stored overnight, 50 μL at 4 °C. Then, serum was obtained by centrifuging the peripheral blood (3000× *g*, 8 min). First, HSA (1 mg/μL) was used to coat 96-well Nunc Maxisorb plates at 4 °C overnight. The plates were washed with 0.05% Tween 20 in PBS five times. Then, 150 μL of 10% fetal bovine serum in PBS were added and incubated at 37 °C for 2 h for nonspecific protein binding. The plates were washed with 0.05% Tween 20 in PBS five times. Then, 100 μL diluted samples (1:2000) were added to the plates and incubated at 37 °C for 2 h. The plates were washed with 0.05% Tween 20 in PBS five times. An amount of 100 μL HRP conjugated anti-mouse IgG1 secondary antibody diluted with 10% FBS in PBS (1:10,000) was added to each well. After that, the plates were incubated at 37 °C for 1 h. The plates were washed with 0.05% Tween 20 in PBS five times. Then, 100 μL of OptEIA 3,3′,5,5′-Tetramethylbenzidine substrate was added to each well and stopped after 25 min with 100 μL of 1 M sulfuric acid. The 450 nm absorbance data was obtained with a Versa Max microplate reader.

### 2.8. Statistical Analysis

All statistical analyses mentioned above were conducted with GraphPad Prism 9. The two-tailed Student’s *t*-test was used to evaluate differences between groups. The measure of one-way analysis of variance (ANOVA) was applied to evaluate differences among all groups. The results were expressed as means ± SE. *p* < 0.05 and *p* < 0.01 stands for statistically significant difference and high statistically significant difference, noted as * *p* < 0.05 and ** *p* < 0.01, respectively.

## 3. Results

### 3.1. FMT and Jujube Powder Recover Antibody and Immune Cells’ Response to HSA Vaccine in ABX-Treated Mice

To reveal the role of gut microbiota in vaccination response, we applied a cocktail of antibiotics to the mice 2 weeks before they received the HSA vaccination (referred to as ABX+HSA hereafter) to observe the vaccine immune response with hampered gut microbiota. Then we applied FMT to the ABX group to restore their gut microbiota. FMT was taken daily from the control mice of the same age and strain to the ABX group for 3 days (referred to as ABX+FMT+HSA); we then determined their vaccination responses. We examined the effectiveness of jujube powder in recovering the gut microbiota of the ABX-treated mice. The ABX-treated mice were fed daily with jujube (800 mg/kg) for a week (referred to as ABX+J+HSA). We also tested the effectiveness of pre-administration of jujube on enhancing the HSA vaccination of the healthy mice (referred to as HSA+J group hereafter). We used 16S rRNA gene sequencing and untargeted metabolomics to assess the above-mentioned gut microbiota and the metabolism related to HSA.

**Figure 1:**
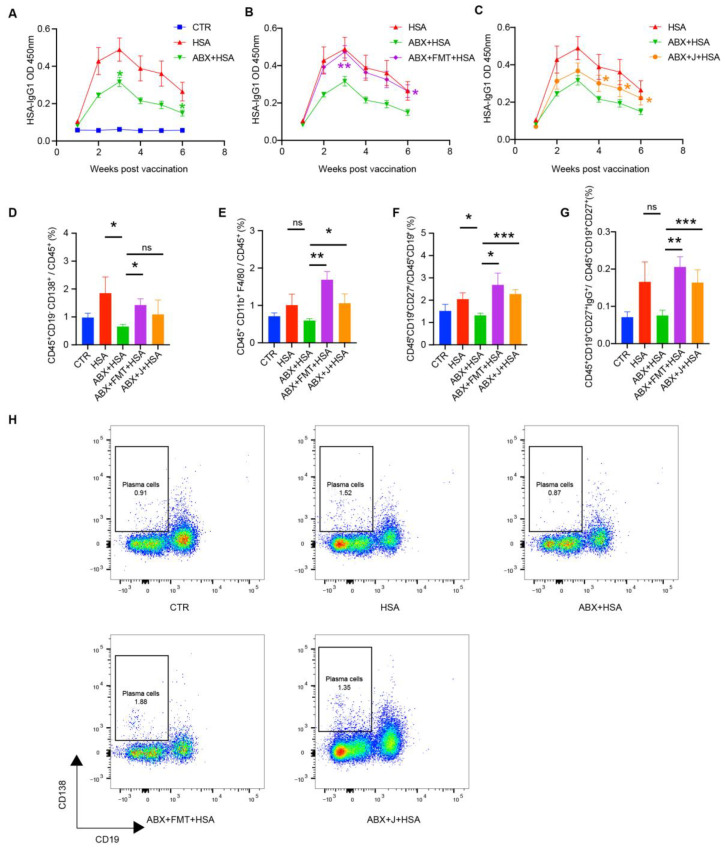
FMT and jujube powder recover antibody and immune cells (plasma cells, macrophages, memory B cells, and IgG memory B cells) of HSA-vaccinated mice models impaired by ABX. (**A**–**C**) HSA-specific IgG1 concentrations in HSA, ABX+HSA group (**A**), ABX+FMT+HSA group (**B**), and ABX+J+HSA group (**C**). (**D**–**G**) The percent of plasma cells (**D**), macrophages (**E**), memory cells (**F**), and IgG memory cells (**G**) in the blood of the CTR, HSA, ABX+HSA, ABX+FMT+HSA, and ABX+J+HSA groups (**H**). Representative flow cytometry results showing the percentage of plasma cells in blood from mice of each group. no significance (ns), *p* > 0.05, * *p* < 0.05, ** *p* < 0.01, *** *p* < 0.001.

As shown in Figure 1A, ABX treatment significantly decreased HSA-specific IgG1 concentrations in the serum of the ABX+HSA mice in weeks 3–6 compared with the HSA group. FMT treatment dramatically increased HSA-specific IgG1 concentrations in the serum, being significantly higher than the ABX+HSA group and closer to the HSA group (Figure 1B). This suggests that a healthy gut microbiota is necessary for antibody response to HSA vaccination. The J group showed a significant increase in HSA-specific IgG1 concentrations in the serum at weeks 4–6 (Figure 1C). The plasma cells and memory B cells, especially IgG memory B cells, in the blood of the ABX+HSA group was much lower. FMT and jujube powder improved those cells (Figure 1D,F–H). Notably, both FMT and jujube powder treatment increased the percent of macrophages in the blood, indicating that macrophages may be the point at which the gut microbiome affects HSA vaccination.

### 3.2. FMT and Jujube Powder Boosted HSA Vaccine Response by Enhancing Macrophages and Plasma Cells Proportions in MLNs of ABX-Treated Mice

**Figure 2:**
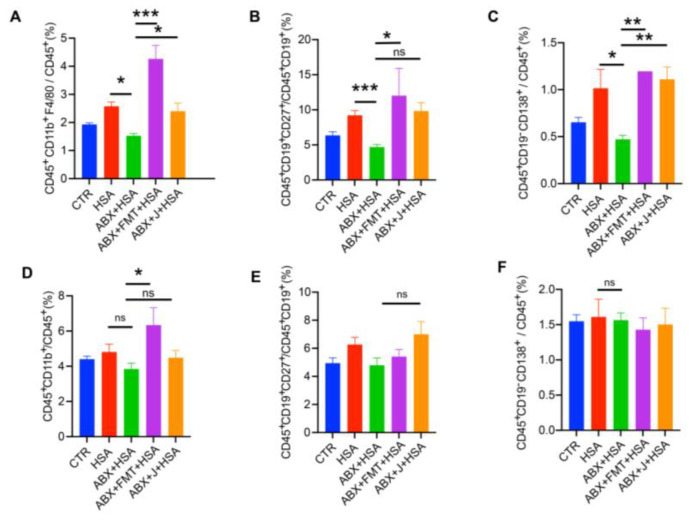
FMT and jujube powder enhanced macrophages, plasma cells, and memory B cells of HSA-vaccinated mice. (**A**–**D**) The proportions of macrophages, memory B cells, plasma cells, and myeloid cells in the MLNs. (**E**,**F**) The proportions of memory B cells and plasma cells in the spleen. no significance (ns), *p* > 0.05, * *p* < 0.05, ** *p* < 0.01, *** *p* < 0.001.

To assess the effects of the MLNs and spleen on HSA vaccination, we examined the immune cell compositions of the MLNs and spleen. As shown in Figure 2A–C, ABX treatment significantly decreased the percentage of macrophages, memory B cells, and plasma cells in the MLNs, whereas FMT enhanced the ratios of macrophages, plasma cells, and memory B cells. Administration of jujube powder also increased macrophages and plasma cells. Interestingly, FMT increased myeloid cells in the spleen, whereas jujube powder had no such effect. Moreover, ABX treatment did not change the proportions of plasma cells and memory B cells in the spleen (Figure 2E,F), which were also not affected by FMT and jujube powder treatment.

### 3.3. Pre-Administration of Jujube Powder Boosted HSA Vaccination

**Figure 3:**
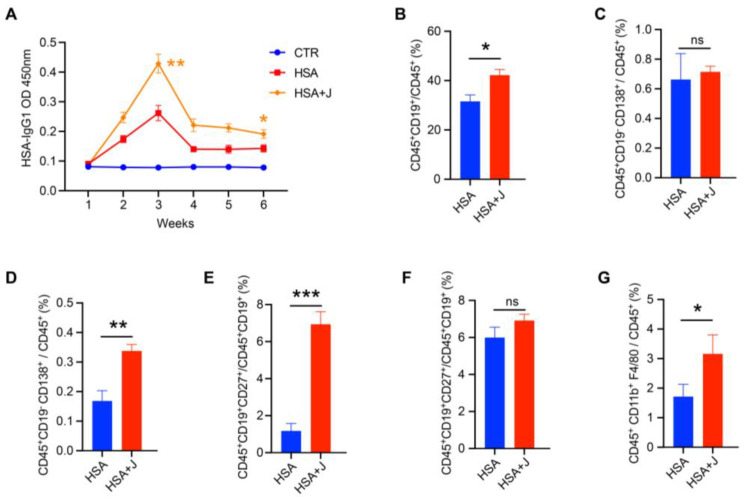
Administrating jujube powder a week in advance enhanced vaccine antibody and immune cell response in mice. (**A**) The concentration of HSA-specific IgG1 antibody in mice serum post-vaccine. (**B**,**C**) The proportions of B cells and plasma cells in the spleens of the HSA and HSA+J groups. (**D**,**E**) The proportions of plasma cells and memory B cells in the peripheral blood of the HSA and HSA+J groups. (**F**) The proportions of memory B cells in the spleens of the HSA and HSA+J groups. (**G**) The proportions of macrophages in the MLNs of the HSA and HSA+J groups. no significance (ns), *p* > 0.05, * *p* < 0.05, ** *p* < 0.01, *** *p* < 0.001.

During the experiment, healthy mice were daily administered jujube (800 mg/kg) a week before vaccination. As shown in Figure 3A, jujube significantly enhanced the peak value of HSA-specific IgG1 antibody response of the serum at 3 weeks, and it still held a high level 6 weeks after HSA vaccination. Figure 3B shows that jujube powder significantly promoted the ratio of B cells in the spleen but had little effect on plasma cells in the spleen (Figure 3C). Plasma cells in the peripheral blood were enhanced (Figure 3D), which was favorable for antibody production during vaccination. In addition, jujube powder markedly improved memory B cells in the peripheral blood, which was more conducive to resisting antigen secondary invasion (Figure 3E). However, jujube powder induced little difference in memory B cells in the spleen (Figure 3F). Macrophages in the MLNs were higher in the J group compared with the control group (Figure 3G), suggesting that jujube powder enhanced B cells’ differentiation to plasma cells and memory B cells by increasing macrophages in the MLNs.

### 3.4. Jujube Altered the Gut Microbiota Diversity and Composition in Mice

**Figure 4:**
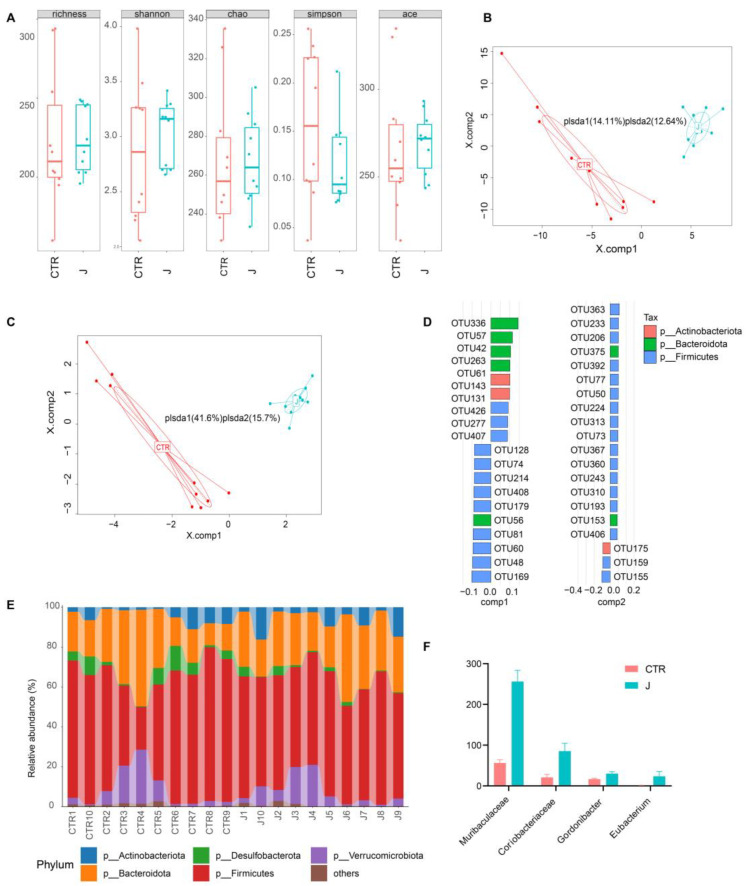
The impact of jujube on gut microbiota diversity and compositions. (**A**) Pre-administration of jujube for a week affected the alpha diversity of gut microbiota according to different indexes. (**B**) Partial least squares discriminant analysis based on OTU abundance. (**C**) Partial least squares discriminant analysis based on the top five representative OTU abundance. (**D**) The critical OTUs conducive to partial least squares discriminant analysis component 1 and component 2. (**E**) The difference in the gut microbiota composition of each mouse at the phylum level. (**F**) The relative abundance of *Muribaculaceae*, *Coriobacteriaceae*, *Gordonibacter*, and *Eubacterium* in the jujube group and the control group.

As shown in Figure 4A, jujube treatment of the healthy mice increased the alpha diversity of the gut microbiota according to the Shannon and Simpson indexes. Figure 4B shows there was a significant difference in the gut microbiota between the control and jujube groups, which was verified by partial least squares discriminant analysis (PLS-DA). The top 5% representative OTUs could repeat those results through PLS-DA (Figure 4C). The most discriminative phyla, which contributed to structural changes in the gut microbiota, are *Actinobacteria*, *Bacteroidota*, and *Firmicutes* (Figure 4D). The *Firmicutes* to *Bacteroidota* ratio decreased in the jujube group compared with the control group (Figure 4E and Figure S2A). At the genus level, jujube significantly increased the abundance of *Muribaculaceae* and *Eubacterium* in the gut microbiota (Figure 4F), which contribute to the metabolism of carbohydrates. Moreover, *Coriobacteriaceae,* association with the metabolism of amino acids, was enriched by administration of jujube, and the relative abundance of *Gordonibacter* was much higher in the jujube group than the control group (Figure 4F), which could induce antigen-presenting cell differentiation.

### 3.5. Jujube Influenced Metabolism Related to Vaccination

**Figure 5:**
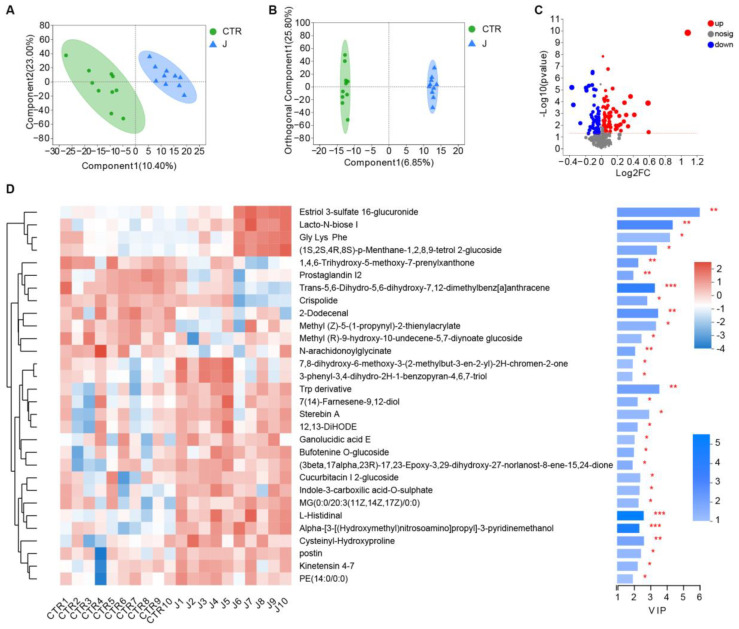
The effect of jujube on the metabolite structure and composition of gut microbiota in mice. (**A**) Partial least squares discriminant analysis based on metabolism variations between the jujube and control groups. (**B**) Orthogonal partial least squares discriminant analysis based on metabolism variations between the jujube and control groups. (**C**) Volcano plot of differences in metabolites between the jujube and control groups (*p* < 0.05); variable importance in the projection of orthogonal partial least squares discriminant analysis (VIP_OPLS-DA > 1). (**D**) Heatmap of important changes in gut microbiota metabolites in each mouse. * *p* < 0.05, ** *p* < 0.01, *** *p* < 0.001.

To investigate the metabolite variations in gut microbiota, we used metabolomics to analyze changes in mice feces a week after jujube administration. As shown in Figure 5A,B, jujube significantly transformed the metabolite structure of the gut microbiota in mice, as confirmed with PLS-DA and orthogonal partial least squares discriminant analysis (OPLS-DA). As shown in Figure 5C, there were 84 metabolite changes in the jujube group compared with the control group (*p* < 0.05, VIP_OPLS-DA > 1). Jujube enhanced the expression of glycine, lysine, and phenylalanine, which might promote B-cell differentiation. Moreover, the increase in lacto-N-biose and estriol 3-sulfate 16-glucuronide suggests improved utilization of carbohydrates in the mice.

### 3.6. KEGG Pathway Enrichment Analysis of Biological Function Induced by Jujube

**Figure 6:**
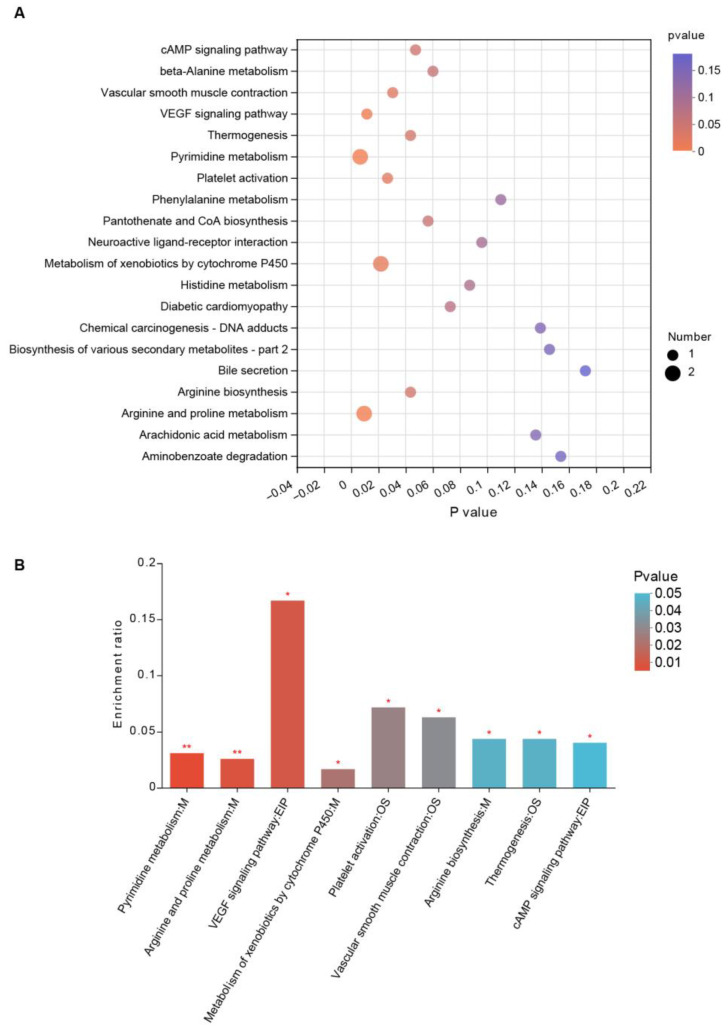
KEGG pathway enrichment analysis reveals the biological significance of gut microbiota metabolites by feeding jujube. (**A**) Bubble chart of KEGG pathway enrichment between the jujube and control groups. (**B**) Significant metabolites of the KEGG pathway enrichment between the jujube and control groups. * *p* < 0.05, ** *p* < 0.01.

We performed KEGG pathway enrichment analysis based on different metabolites caused by jujube to evaluate their biological significance. As shown in Figure 6A, there were several differential KEGG pathways between the jujube and control groups. Jujube administration enhanced arginine and proline metabolism (Figure 6B), revealing the possible mechanism of promoting macrophages in MLNs (Figure 3G). Moreover, jujube upregulated pyrimidine metabolism, which was associated with activating the immune system. Metabolism of xenobiotics by cytochrome P450 was boosted by jujube, which was conducive to antioxidation.

## 4. Discussion

The present study confirmed that the gut microbiota plays an indispensable role in HSA vaccination, in that ABX treatment significantly impaired immunity induced by vaccines (Figure 1A,D–H), while FMT increased the HSA-IgG1 level in serum (Figure 1B) and enhanced macrophages in MLNs (Figure 2A), plasma cells, and memory B cells in the peripheral blood (Figure 1D–F). Lynn et al. [6] found that recovering the commensal microbiota via FMT restored the antibody response impaired by antibiotics in infant mice. The administration of jujube powder resecured poor vaccine immune response induced by ABX treatment; increased the concentration of HSA-IgG1 in serum (Figure 1C); and increased the percentage of plasma cells and memory B cells in the peripheral blood (Figure 1D) and macrophages, memory B cells, and plasma cells in the MLNs (Figure 2A–C). The administration of jujube powder did not improve myeloid cells in comparison with the ABX+FMT+HSA group (Figure 2D). Moreover, pre-administration of jujube powder boosted HSA vaccine immune response in healthy mice, including increasing the HSA-IgG1 level in the serum (Figure 3A), enhancing B cells and plasma cells in the spleen, and inducing differentiation of plasma cells and memory B cells in the peripheral blood and macrophages in the MLNs (Figure 3B–G) at week 6 post vaccine.

The study demonstrated that jujube powder increased the alpha diversity of gut microbiota (Figure 4A) and changed the composition of gut microbiota according to PLS-DA (Figure 4B–E). At the genus level, jujube significantly increased the abundance of *Muribaculaceae* [26,27] and *Eubacterium* [28] in gut microbiota, which contribute to the metabolism of carbohydrates. Moreover, *Coriobacteriaceae,* associated with metabolism of amino acids [22], was enriched by the administration of jujube, and the abundance of *Gordonibacter* was much higher in the jujube group than the control group (Figure 4F), which could induce antigen-presenting cell differentiation [21]. Metabolomics revealed that jujube altered the composition of gut microbiota metabolites according to PLS-DA and OPLS-DA (Figure 5A,B) and enhanced the expression of glycine, lysine, and phenylalanine, which might promote B-cell differentiation (Figure 5C,D). Moreover, the increase in the lacto-N-biose [29,30] and estriol 3-sulfate 16-glucuronide [31] suggests improved utilization of carbohydrates in the mice. KEGG pathway enrichment suggests that jujube administration enhanced arginine and proline metabolism (Figure 6A,B), revealing the possible mechanism of promoting macrophages in MLNs [32,33,34] (Figure 3G). Moreover, jujube upregulated pyrimidine metabolism [35], which was associated with activating the immune system.

Interestingly, metabonomic data suggested that the administration of jujube could boost xenobiotics with cytochrome P450, which is conducive to antioxidation. Platelet activation was also enhanced by the administration of jujube, deserving further study in autoimmune diseases. It should be noted, however, that the current study was performed in mice models. Considering the interspecies and individual diversities, the results may not be fully applicable to humans, which need further study in clinical trials.

## 5. Conclusions

We observed that vaccine immune responses were impaired in HSA-vaccinated mice with ABX treatment. FMT recovered the HSA-specific IgG1 concentration in the serum by increasing the proportions of macrophages in the MLNs and plasma cells in the peripheral blood. Meanwhile, the administration of jujube powder induced higher HSA-specific IgG1 concentration in the serum than the ABX+HSA group without improving myeloid cells in comparison with the ABX+FMT+HSA group. Moreover, pre-administration of jujube powder boosted vaccine immune response in healthy mice. Jujube powder improved the proportion of macrophages in the MLNs, B cells in the spleen, and plasma cells in the peripheral blood, which are responsible for producing HSA-specific IgG1. The administration of jujube powder enhanced the percentage of memory B cells, which are conducive to resisting antigen-secondary invasion. Jujube powder raised the alpha diversity of the gut microbiota. Especially, *Eubacterium* enriched by jujube powder could induce antigen-presenting cell differentiation, and *Coriobacteriaceae* are associated with the metabolism of amino acids. Through KEGG pathway enrichment analysis, we found that jujube administration enhanced arginine, proline, and pyrimidine metabolism, revealing the possible mechanism of promoting macrophages and plasma cells. All these results suggest the utilization of jujube powder as prebiotics to recover the vaccine immune response of ABX-treated mice and enhance that of healthy mice by regulating gut microbiota.

## Figures and Tables

**Table 1:** Cell-marker schemes for identification of cell populations.

Cells	Markers [25]
B cells	CD45^+^ CD19^+^
Plasma cells	CD45^+^ CD19^−^ CD138^+^
Memory B cells	CD45^+^ CD19^+^ CD27^+^
IgG memory B cells	CD45^+^ CD19^+^ CD27^+^ IgG^+^
Macrophages	CD45^+^ CD11b^+^ F4/80^+^
Myeloid	CD45^+^ CD11b^+^

**Table 2:** The fluorophore conjugates and manufacture of cell markers.

Markers	Conjugate	Manufacture
CD45	PerCP-Cy5.5	BioLegend
CD19	Alexa Fluor 700	BioLegend
CD138	PE-Cy7	BioLegend
CD27	APC	BioLegend
CD11b	FITC	BioLegend
F4/80	Pacific Blue	BioLegend
IgG	PE	BioLegend

## Data Availability

The raw sequences of 16S rRNA are acquirable after 18 June 2025 (https://www.ncbi.nlm.nih.gov/) through project ID PRJNA942135.

## Supplementary Materials

The following supporting information can be downloaded at: https://www.mdpi.com/article/10.3390/nu15081955/s1, Figure S1: The scheme of experimental procedures; Figure S2: The abundance of *Firmicutes* and *Bacteroidota* in the control and jujube groups; Table S1: Relevant statistics depend on the statistical approach.
