# Supplementary material for: Boosting HSA Vaccination with Jujube Powder Modulating Gut Microbiota Favorable for Arginine Metabolism
Source: Nutrients. 2023 Apr 19;15(8):1955. doi: 10.3390/nu15081955 (PMC10142099; doi:10.3390/nu15081955)
Supplement: Supplementary file 1 [file nutrients-15-01955-s001.zip › nutrients-2328799-supplementary-updated.pdf]

Supporting Information

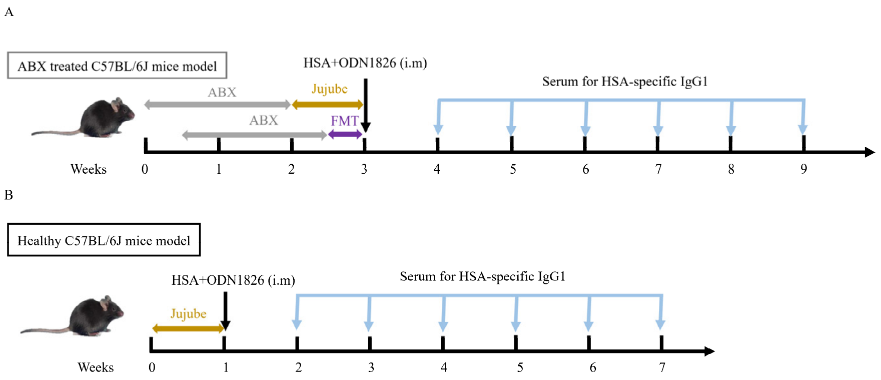

Figure S1. The scheme of experimental procedures. (A) In the antibiotic cocktail (ABX) treated mice model, firstly, mice were fed water containing ABX (1 g/L ampicillin, 1 g/L neomycin, 1 g/L metronidazole, and 0.5 g/L vancomycin) for 2 weeks. Then, mice in ABX+J+HSA group were daily oral administration of jujube powder (800 mg/kg) for a week. Mice in ABX+FMT+HSA group were daily fecal microbiota transferred (FMT) from the fecal of same strain and age mice for 3 days. After that, mice were intramuscular (i.m) injected with 90 μg human serum albumin (HSA) and 5 μg oligodeoxynucleotide 1826 (ODN1826) dissolved in 100μL saline solution. The mice in control (CTR) group were i.m injected with equal volume saline solution. Each of CTR, HSA, ABX+HSA, ABX+J+HSA and ABX+FMT+HSA group had 8 mice. (B) In the healthy mice model, mice in J group were daily pre-administration of jujube powder (800 mg/kg) for a week, and they were i.m injected with HSA (90 μg) and ODN1826 (5 μg) dissolved in 100 μL saline solution. Each of CTR, J, HSA, and HSA+J group had 10 mice. Mice were collected 30uL peripheral blood with 1.5% isoflurane every week through retro-orbital sinus puncture into capillary tubes.

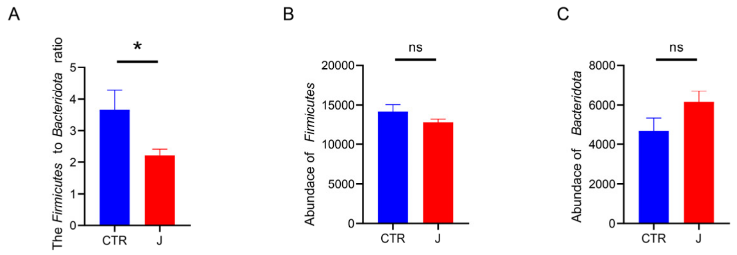

Figure S2. The abundance of *Fimicutes* and *Bacteridota* in control and jujube group. (A) The ratio of *Fimicutes* and *Bacteridota* in CTR and J group. (B) The abundance of *Fimicutes* CTR and J group. (C) The abundance of *Bacteridota* CTR and J group. no significance (ns) , * $p < 0.05$.

Table S1. Relevant statistics depend on the statical approach

Figure	Compared data	T test		Anova
		T value	df value	F value
1A	ABX+HSA vs ABX (week3)	2.57	9.13	
	ABX+HSA vs ABX (week6)	2.97	7.17	
1B	ABX+HSA+FMT vs ABX+HSA (week3)	3.47	10	
	ABX+HSA+FMT vs ABX+HSA (week6)	2.5	11	
1C	ABX+HSA+J vs ABX+HSA (week4)	2.63	10.13	
	ABX+HSA+J vs ABX+HSA (week5)	2.71	7.57	
	ABX+HSA+J vs ABX+HSA (week6)	2.88	5.24	
1D	ABX+HSA vs the other groups			2.58
1E	ABX+HSA vs the other groups			3.5
1F	ABX+HSA vs the other groups			4.34
1G	ABX+HSA vs the other groups			3.24
2A	ABX+HSA vs the other groups			16.18
2B	ABX+HSA vs the other groups			3.25
2C	ABX+HSA vs the other groups			4.3
2D	ABX+HSA vs the other groups			3.66
2E	ABX+HSA vs the other groups			2.67
2F	ABX+HSA vs the other groups			4.16
3A	HSA+J vs HSA(week3)	3.35	11	
	HSA+J vs HSA(week6)	2.59	9.57	
3B	HSA+J vs HSA	3.02	17	
3C	HSA+J vs HSA	0.32	14	
3D	HSA+J vs HSA	4.12	14	
3E	HSA+J vs HSA	4.01	17	
3F	HSA+J vs HSA	1.41	14	
3G	HSA+J vs HSA	2.84	12	
